# Harringtonine Attenuates Extracellular Matrix Degradation, Skin Barrier Dysfunction, and Inflammation in an In Vitro Skin Aging Model

**DOI:** 10.3390/cimb47080642

**Published:** 2025-08-10

**Authors:** Sullim Lee, Sanghyun Lee

**Affiliations:** 1 Department of Life Science, College of Bio-Nano Technology, Gachon University, Seongnam 13120, Republic of Korea; sullimlee@gachon.ac.kr; 2 Department of Plant Science and Technology, Chung-Ang University, Anseong 17546, Republic of Korea; 3 Natural Product Institute of Science and Technology, Anseong 17546, Republic of Korea

**Keywords:** harringtonine, TNF-α/IFN-γ, skin aging, inflammation, ECM, skin barrier

## Abstract

With the growing interest in natural strategies for preventing skin aging, plant-derived compounds are being actively investigated for their potential protective effects against skin inflammation and extracellular matrix (ECM) degradation. In this study, we explored the anti-aging and anti-inflammatory effects of harringtonine, an alkaloid isolated from *Cephalotaxus harringtonia*, in normal human epidermal keratinocytes (NHEKs) under inflammatory stress induced by tumor necrosis factor-alpha (TNF-α) and interferon-gamma (IFN-γ). Harringtonine significantly suppressed the expression of matrix metalloproteinases (*MMP)-1*, *MMP-2*, and *MMP-9* and restored the expression of collagen synthesis-related genes [collagen type I alpha 1 chain (*COL1A1*), collagen type I alpha 2 chain (*COL1A2*), and collagen type IV alpha 1 chain *COL4A1*)], indicating its protective role in ECM degradation. Additionally, harringtonine improved the expression of skin barrier-related genes, such as serine peptidase inhibitor kazal type 5 (*SPINK5*), loricrin (*LOR*), quaporin-3 (*AQP3*), filaggrin (*FLG*), and keratin 1 (*KRT1*) although it had no significant effect on involucrin (*IVL*). Harringtonine also markedly reduced the production of pro-inflammatory cytokines [interleukin (IL)-1β, IL-6, and IL-8] and inflammatory mediators, including prostaglandin E_2_ (PGE_2_), cyclooxygenase-2 (COX-2), and nitric oxide (NO). Our findings suggest that harringtonine may serve as a promising natural compound for mitigating skin aging and inflammation through multi-targeted modulation of ECM remodeling, skin barrier function, and inflammatory response.

## 1. Introduction

Reactive oxygen species (ROS) are central mediators of oxidative stress, contributing to the onset and progression of various human diseases through cellular and molecular damage [1,2]. Mitochondrial oxidative phosphorylation is the primary source of ROS under physiological conditions, where electron leakage from the electron transport chain generates ROS through interactions with molecular oxygen [3]. To maintain redox homeostasis, cells produce antioxidant enzymes such as superoxide dismutase (SOD), catalase, and glutathione peroxidase, which mitigate oxidative damage by scavenging ROS [4]. However, excessive ROS production or insufficient antioxidant defense results in oxidative stress, a pathological state implicated in neurodegenerative diseases, cardiovascular disorders, and skin aging.

The skin is particularly susceptible to oxidative stress owing to continuous exposure to environmental insults, including ultraviolet (UV) radiation, air pollutants, and chemical oxidants [5]. Skin aging is classified into intrinsic and extrinsic types of aging. Intrinsic aging reflects the natural decline in cellular function and accumulation of ROS during normal metabolism, whereas extrinsic aging is driven by environmental stressors, most notably UV radiation and pollution [6]. ROS accumulation in the skin promotes oxidative damage to key ECM components, such as collagen and elastin, resulting in their fragmentation and crosslinking, ultimately compromising skin structure and elasticity [4].

Matrix metalloproteinases (MMPs), particularly MMP-1, MMP-2, and MMP-9, are strongly induced by oxidative stress and inflammatory stimuli, and are responsible for degrading ECM proteins, including collagen and elastin, thus accelerating dermal aging processes such as wrinkle formation and skin laxity [7]. The collagen genes *COL1A1*, *COL1A2*, *COL3A1*, and *COL4A1* encode structural proteins essential for maintaining dermal integrity, and their downregulation is a hallmark of aged or damaged skin [8]. Similarly, hyaluronan synthases (*HAS-1*, *HAS-2*, and *HAS-3*), which are critical for hyaluronic acid production and skin hydration, are often suppressed under oxidative or inflammatory conditions, contributing to skin dryness and reduced elasticity [9].

Skin barrier-related proteins, including *SPINK5*, *LOR*, *IVL*, *AQP3*, *FLG*, and *KRT1*, play essential roles in maintaining epidermal barrier function and hydration. Dysregulation of these proteins is associated with impaired barrier integrity, increased transepidermal water loss, and heightened susceptibility to external stressors [10].

Pro-inflammatory cytokines, such as IL-1β, IL-6, and IL-8, are major mediators of skin inflammation and aging. These cytokines promote the recruitment of immune cells, enhance ROS production, and stimulate MMP expression, establishing a vicious cycle of inflammation and tissue degradation [11]. Notably, TNF-α and IFN-γ further exacerbate oxidative stress and inflammation by upregulating MMPs and inflammatory cytokines while simultaneously impairing ECM and skin barrier protein expression [12].

Taken together, these molecular factors, including MMPs, collagen genes, HAS enzymes, barrier-related proteins, and cytokines, form an intricate network that regulates oxidative stress, ECM integrity, skin barrier function, and inflammatory responses. Thus, they are critical targets for investigating the efficacy of antioxidant and anti-inflammatory agents in in vitro skin cell models.

**Figure 1:**
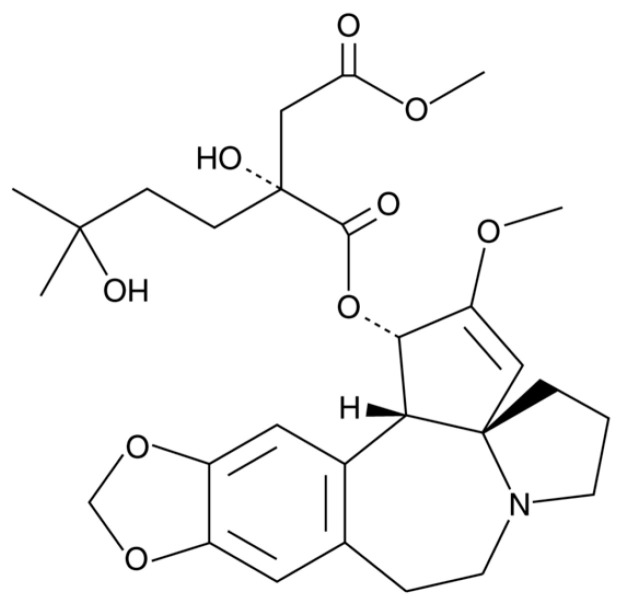
Chemical structure of harringtonine (HTN).

Among various plant-derived bioactive compounds, harringtonine (Figure 1, Supplementary Materials), an alkaloid isolated from *Cephalotaxus harringtonia*, has gained attention for its anti-skin aging activity in human dermal fibroblasts [13]. The purity of harringtonine was 98.29% by HPLC analysis from Natural Product Institute of Science and Technology, Anseong, Korea. However, its potential effects on skin aging, particularly under inflammatory stress conditions induced by the combined stimulation of TNF-α and interferon-gamma (IFN-γ), remain largely unexplored. Given the pivotal roles of TNF-α and IFN-γ in promoting skin inflammation, extracellular matrix (ECM) degradation, and disruption of the skin barrier, the identification of novel compounds that can effectively mitigate these pathological processes is of significant therapeutic interest. In particular, compounds that exhibit multi-target effects, such as modulating ECM remodeling, restoring skin barrier function, and regulating inflammatory cytokine production, may serve as promising candidates for the prevention or treatment of skin aging. In this context, the potential of harringtonine as a protective agent against skin damage warrants further investigation.

## 2. Materials and Methods

### 2.1. Cell Culture

NHEKs used in this study were obtained from PromoCell GmbH (Heidelberg, Germany). The cells were cultured in Dulbecco’s modified Eagle’s medium (DMEM; Corning, Manassas, VA, USA) supplemented with 10% fetal bovine serum (FBS; Atlas, Fort Collins, CO, USA) and 1% penicillin–streptomycin (Gibco, Grand Island, NY, USA) to provide the essential nutrients and antibiotics for optimal cell growth. The culture medium was replaced every two to three days to maintain healthy cell conditions and prevent nutrient depletion or the accumulation of waste. The cells were maintained at 37 °C in a humidified incubator with 5% CO_2_ to ensure an environment suitable for keratinocyte proliferation and viability. All experiments were performed using cells at passage numbers below 12.

### 2.2. Real-Time PCR

NHEKs were seeded into flat-bottomed 6-well plates at a density of 3 × 10^5^ cells per well and incubated for 24 h at 37 °C in a humidified atmosphere containing 5% CO_2_ to allow proper attachment. After incubation, the medium was replaced with serum-free medium, and the cells were subjected to serum starvation for an additional 24 h to synchronize their physiological state. Following serum starvation, the cells were pretreated with harringtonine at concentrations of 3, 10, and 30 µM for 1 h, followed by treatment with TNF-α and IFN-γ (20 ng/mL each) and incubation for an additional 4 or 24 h.

Total RNA was extracted from the cells using an RNeasy Mini Kit (Qiagen, Germantown, MD, USA) according to the manufacturer’s protocol. The quality and concentration of the isolated RNA were assessed using spectrophotometry, and equal amounts of RNA from each sample were used for complementary DNA (cDNA) synthesis to ensure consistency across the experimental groups. cDNA was synthesized from the extracted RNA using the RevertAid First Strand cDNA Synthesis Kit (Thermo Fisher Scientific, Eugene, OR, USA), following the manufacturer’s instructions. Quantitative real-time PCR (qPCR) was performed using PowerUp SYBR Green Master Mix (Thermo Fisher Scientific, Austin, TX, USA) to measure the mRNA expression levels of the target genes. Amplification reactions were performed using a QuantStudio 3 Real-Time PCR System (Applied Biosystems, Thermo Fisher Scientific, Foster City, CA, USA). The specific primer sequences used for amplifying the target genes are listed in Table 1. The qPCR cycling conditions were as follows: an initial incubation at 50 °C for 2 min to activate the uracil-DNA glycosylase (UDG) enzyme, followed by denaturation at 95 °C for 10 min to activate DNA polymerase and denature the template strands. This was followed by 40 amplification cycles, each consisting of denaturation at 95 °C for 15 s and annealing/extension at 60 °C for 1 min. A final dissociation step was included to confirm the specificity of the amplification, with sequential incubation at 95 °C for 15 s, 60 °C for 1 min, and 95 °C for 15 s to generate a melt curve.

### 2.3. Enzyme-Linked Immunosorbent Assay (ELISA)

NHEKs were seeded into flat-bottomed 48-well plates at a density of 2 × 10^4^ cells per well. The cells were incubated for 24 h at 37 °C in a humidified incubator with 5% CO_2_ to allow proper attachment and recovery. After incubation, the medium was removed and replaced with serum-free medium to induce serum starvation, which was maintained for 24 h to minimize the influence of external growth factors and synchronize the cells. Following starvation, the cells were treated with the test samples for 1 h to allow sufficient interaction between the samples and the cells. After sample treatment, the cells were exposed to TNF-α and IFN-γ at a concentration of 20 ng/mL and incubated for another 24 h to trigger an inflammatory response. At the end of the treatment period, culture supernatants were collected, and the secretion levels of IL-1β, IL-6, IL-8, PGE_2_, and COX-2 were measured using enzyme-linked immunosorbent assay (ELISA) kits (R&D Systems, Minneapolis, MN, USA), following the manufacturer’s instructions. The optical density was measured at 450 nm using a microplate reader (SPARK 10M, Tecan Group Ltd., Männedorf, Switzerland) to quantify the protein concentrations.

### 2.4. Measurement of NO Production

NHEKs were seeded into flat-bottomed 96-well plates at a density of 1 × 10^4^ cells per well and incubated for 24 h at 37 °C in a humidified atmosphere containing 5% CO_2_ to allow proper attachment. After incubation, the medium was replaced with serum-free medium, and the cells were subjected to serum starvation for an additional 24 h to synchronize their physiological state. Following serum starvation, the cells were treated with harringtonine for 1 h to evaluate its effects. After this treatment, the cells were stimulated with TNF-α and IFN-γ at a final concentration of 20 ng/mL and incubated for another 24 h.

At the end of the treatment period, the culture supernatants were collected, and the secretion levels of Nitric Oxide (NO) were measured using Griess reagent (Promega Corporation, Madison, WI, USA). Optical density was measured at 540 nm using a microplate reader (SPARK 10M, Tecan Group Ltd., Männedorf, Switzerland) to quantify NO concentrations. NO concentrations (ng/mL) for each treatment were calculated and compared with sodium nitrite (NaNO_2_; Sigma-Aldrich, St. Louis, MO, USA) standard.

### 2.5. Statistical Analysis

All data are presented as mean ± standard error of the mean (SEM) based on at least three independent experiments. Statistical significance was analyzed using one-way analysis of variance (ANOVA) to determine whether there were significant differences between the experimental groups. Tukey’s multiple-comparison test was used as a post hoc analysis to identify specific differences between groups. All statistical analyses were performed using GraphPad Prism software (version 10.2.0; GraphPad Software, San Diego, CA, USA). A *p*-value of less than 0.05 was considered statistically significant throughout the study.

## 3. Results

### 3.1. Effect of Harringtonine on Skin Aging-Related Genes in TNF-α/IFN-γ-Stimulated NHEKs

Our previous study demonstrated that *C. harringtonia* extract and its active components exhibit anti-aging effects. In particular, harringtonine was identified as a potential active ingredient for anti-aging due to its ability to inhibit MMP-1 protein secretion and reduce collagen degradation. Therefore, in this study, we investigated the effects of harringtonine on the mRNA expression of skin aging-related genes in NHEKs stimulated with TNF-α and IFN-γ.

**Figure 2:**
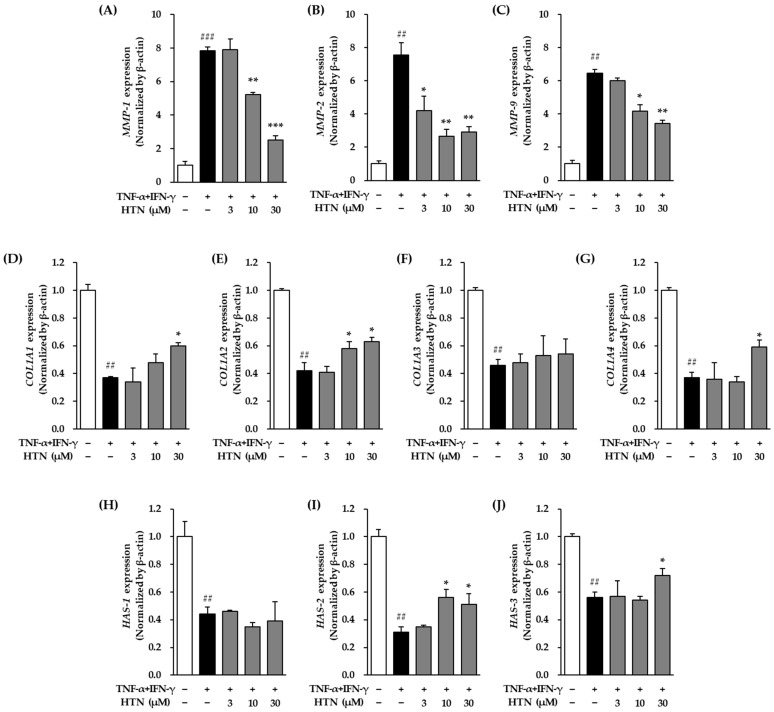
Effects of harringtonine (HTN) on *MMP-1*, *MMP-2*, *MMP-9*, *COL1A1*, *COL1A2*, *COL3A1*, *COL4A1*, *HAS-1*, *HAS-2*, and *HAS-3* mRNA expression in TNF-α/IFN-γ-stimulated normal human epidermal keratinocyte (NHEKs). NHEKs were seeded at 2 × 10^4^ cells/well in 48-well plates and incubated for 24 h. After serum starvation for 24 h, the cells were pretreated with harringotnine for 1 h, followed by stimulation with 20 ng/mL TNF-α/IFN-γ for 24 h. mRNA expression levels of *MMP-1* (**A**), *MMP-2* (**B**), *MMP-9* (**C**), *COL1A1* (**D**), *COL1A2* (**E**), *COL3A1* (**F**), *COL4A1* (**G**), *HAS-1* (**H**), *HAS-2* (**I**), and *HAS-3* (**J**) were quantified by real-time PCR. Data are shown as fold change relative to the TNF-α/IFN-γ-treated group and expressed as mean ± SEM from two independent experiments. ^##^ *p* < 0.01, ^###^ *p* < 0.001 vs. vehicle control; * *p* < 0.05, ** *p* < 0.01, *** *p* < 0.001 vs. TNF-α/IFN-γ-treated group.

The mRNA expression levels of matrix metalloproteinases (MMPs) were markedly altered in TNF-α/IFN-γ-stimulated NHEKs cells. Specifically, TNF-α/IFN-γ treatment (20 ng/mL for 12 h) led to a significant increase in MMP-1 expression, reaching 7.85 ± 0.21-fold relative to the untreated control group (*p* < 0.001) (Figure 2A), whereas harringtonine co-treatment significantly attenuated this induction, reducing the expression level to 5.21 ± 0.14-fold at 10 μM (*p* < 0.01) and 2.50 ± 0.28-fold at 30 μM (*p* < 0.001). A similar trend was observed for MMP-2, with TNF-α/IFN-γ significantly elevating its mRNA expression to 7.54 ± 0.76-fold compared to the control group (*p* < 0.01), and harringtonine effectively lowering it to 4.21 ± 0.87-fold at 3 μM (*p* < 0.05), 2.65 ± 0.42-fold at 10 μM (*p* < 0.01) and 2.91 ± 0.32-fold at 30 μM (*p* < 0.01)(Figure 2B). In the case of MMP-9, TNF-α/IFN-γ stimulation resulted in a pronounced increase in its mRNA expression to 6.44 ± 0.24-fold (*p* < 0.001), which was significantly suppressed by harringtonine treatment, reducing its level to 4.18 ± 0.37-fold at 10 μM (*p* < 0.05) and 3.44 ± 0.18-fold at 30 μM (*p* < 0.01) compared to that in the TNF-α/IFN-γ-only group (*p* < 0.01) (Figure 2C).

Regarding collagen-related genes, TNF-α/IFN-γ treatment led to a notable reduction in *COL1A1* mRNA expression, decreasing it to 0.37 ± 0.01-fold relative to the control group (*p* < 0.01) (Figure 2D), whereas co-treatment with harringtonine markedly restored its expression to 0.48 ± 0.06-fold at 10 μM (not significant) and 0.60 ± 0.02-fold at 30 μM (*p* < 0.05). Similarly, *COL1A2* expression was significantly downregulated by TNF-α/IFN-γ, dropping to 0.42 ± 0.06-fold compared to the control (*p* < 0.01), whereas harringtonine effectively reversed this suppression, restoring its level to 0.58 ± 0.05-fold at 10 μM (*p* < 0.05) and 0.63 ± 0.03-fold at 30 μM (*p* < 0.05) (Figure 2E). In addition, TNF-α/IFN-γ markedly reduced *COL3A1* expression to 0.46 ± 0.04-fold (*p* < 0.001), but harringtonine co-treatment increased its expression to 0.48 ± 0.14-fold at 10 μM (not significant) and 0.63 ± 0.03-fold at 30 μM (not significant) relative to the TNF-α/IFN-γ-treated group (Figure 2F). The expression of *COL4A1* also followed this pattern, with TNF-α/IFN-γ reducing its mRNA levels to 0.37 ± 0.04-fold (*p* < 0.01) and 30 μM harringtonine restoring it to 0.59 ± 0.05-fold (*p* < 0.05) (Figure 2G).

Furthermore, the expression levels of hyaluronic acid synthase (*HAS*) genes were significantly affected by TNF-α/IFN-γ stimulation. *HAS-1* expression was markedly suppressed, showing a decrease to 0.44 ± 0.05-fold compared to the control (*p* < 0.05); however, harringtonine treatment did not significantly alter its expression compared to the TNF-α/IFN-γ-only group (Figure 2H). Similarly, TNF-α/IFN-γ significantly downregulated *HAS-2* and *HAS-3* mRNA expression, decreasing to 0.31 ± 0.04-fold and 0.56 ± 0.04-fold, respectively (*p* < 0.01 for both) (Figure 2I,J). However, harringtonine co-treatment restored *HAS-2* levels to 0.56 ± 0.06-fold at 10 μM (*p* < 0.05) and 0.51 ± 0.08-fold at 30 μM (*p* < 0.05) compared to the TNF-α/IFN-γ-treated group (Figure 2I). Harringtonine co-treatment restored *HAS-3* expression levels to 0.72 ± 0.05-fold at 30 μM (*p* < 0.05) compared to the TNF-α/IFN-γ-treated group (Figure 2J).

### 3.2. Effect of Harringtonine on the mRNA Expression of Skin Barrier-Related Genes in TNF-α/IFN-γ-Stimulated NHEKs

The mRNA expression levels of skin barrier-related genes, including *SPINK5*, *LOR*, *IVL*, *AQP3*, *FLG*, and *KRT1*, were quantitatively analyzed to investigate the effects of harringtonine on skin barrier function in TNF-α/IFN-γ-stimulated NHEKs.

**Figure 3:**
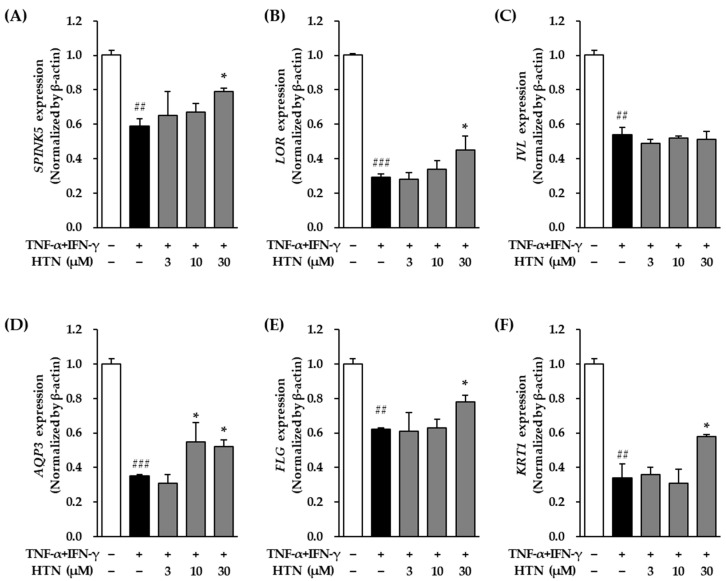
Effects of harringtonine (HTN) on *SPINK5*, *LOR*, *IVL*, *AQP3*, *FLG*, and *KRT1* mRNA expression in TNF-α/IFN-γ-stimulated normal human epidermal keratinocyte (NHEKs). NHEKs were seeded at 2 × 10^4^ cells/well in 48-well plates and incubated for 24 h. After serum starvation for 24 h, the cells were pretreated with harringotnine for 1 h, followed by stimulation with 20 ng/mL TNF-α/IFN-γ for 24 h. mRNA expression levels of *SPINK5* (**A**), *LOR* (**B**), *IVL* (**C**), *AQP3* (**D**), *FLG* (**E**), and *KRT1* (**F**) were quantified by real-time PCR. Data are shown as fold change relative to the TNF-α/IFN-γ-treated group and expressed as mean ± SEM from two independent experiments. ^##^ *p* < 0.01, ^###^ *p* < 0.001 vs. vehicle control; * *p* < 0.05 vs. TNF-α/IFN-γ-treated group.

TNF-α/IFN-γ stimulation (20 ng/mL for 12 h) significantly decreased *SPINK5* mRNA expression compared to the untreated control group, with the expression level reduced to 0.59 ± 0.04-fold (*p* < 0.01) (Figure 3A). Co-treatment with harringtonine significantly restored *SPINK5* mRNA expression, increasing it to 0.79 ± 0.02-fold at 30 μM relative to the TNF-α/IFN-γ-treated group (*p* < 0.05).

Similarly, *LOR* mRNA expression was markedly reduced following TNF-α/IFN-γ treatment, with expression levels decreasing to 0.29 ± 0.02-fold compared to the control group (*p* < 0.001) (Figure 3B). Harringtonine co-treatment effectively reversed this reduction, significantly restoring *LOR* mRNA expression to 0.45 ± 0.08-fold at 30 μM relative to the TNF-α/IFN-γ-treated group (*p* < 0.05).

TNF-α/IFN-γ stimulation also significantly decreased *IVL* mRNA expression, with levels dropping to 0.54 ± 0.04-fold compared to the control group (*p* < 0.01) (Figure 3C). However, harringtonine treatment did not result in a significant change in *IVL* mRNA expression relative to the TNF-α/IFN-γ-treated group.

In the case of *AQP3*, mRNA expression was also significantly suppressed by TNF-α/IFN-γ stimulation, with the expression level reduced to 0.35 ± 0.01-fold compared to the control (*p* < 0.001) (Figure 3D). Harringtonine co-treatment markedly increased *AQP3* mRNA expression, restoring it to 0.55 ± 0.11-fold at 10 μM and 0.52 ± 0.04-fold at 30 μM relative to the TNF-α/IFN-γ-treated group (for both *p* < 0.05).

Similarly, *FLG* mRNA expression was significantly decreased following TNF-α/IFN-γ treatment, with expression levels reduced to 0.62 ± 0.01-fold compared to the control group (*p* < 0.01) (Figure 3E). Harringtonine co-treatment significantly restored *FLG* mRNA expression, increasing it to 0.78 ± 0.04-fold at 30 μM relative to the TNF-α/IFN-γ-treated group (*p* < 0.05).

Finally, *KRT1* mRNA expression was markedly suppressed by TNF-α/IFN-γ stimulation, with levels decreasing to 0.34 ± 0.08-fold compared to the control group (*p* < 0.01) (Figure 3F). Co-treatment with harringtonine effectively alleviated this reduction, restoring *KRT1* mRNA expression to 0.58 ± 0.01-fold at 30 μM relative to the TNF-α/IFN-γ-treated group (*p* < 0.05).

### 3.3. Effect of Harringtonine on the Secretion and mRNA Expression of Inflammatory Cytokines IL-1β, IL-6, and IL-8 in TNF-α/IFN-γ-Stimulated NHEKs

The secretion levels and mRNA expression of the pro-inflammatory cytokines IL-1β, IL-6, and IL-8 were quantitatively analyzed to evaluate the anti-inflammatory effects of harringtonine in TNF-α/IFN-γ-stimulated NHEKs.

**Figure 4:**
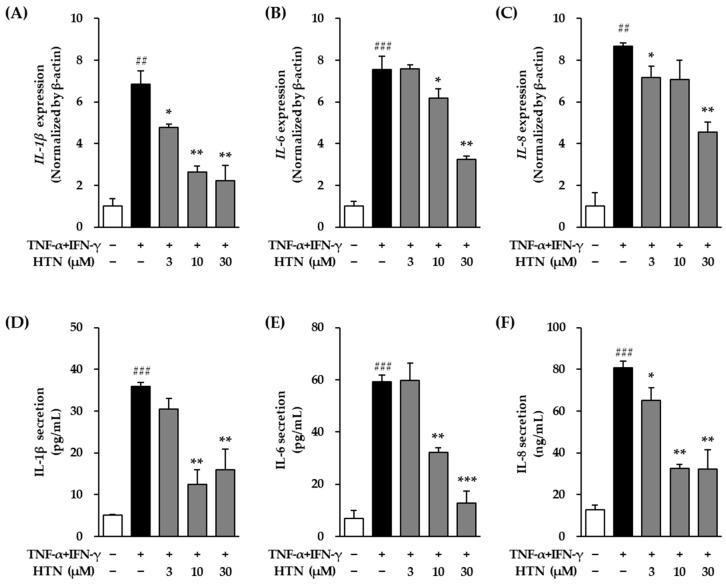
Effects of harringtonine (HTN) on protein secretion and mRNA expression of IL-1β, IL-6, and IL-8 in normal human epidermal keratinocyte (NHEKs). NHEKs were seeded at 2 × 10^4^ cells/well in 48-well plates and incubated for 24 h. After serum starvation for 24 h, the cells were pretreated with harringotnine for 1 h, followed by stimulation with 20 ng/mL TNF-α/IFN-γ for 24 h. mRNA expression levels of *IL-1β* (**A**), *IL-6* (**B**), and *IL-8* (**C**) and protein levels of IL-1β (**D**), IL-6 (**E**), and IL-8 (**F**) were quantified by real-time PCR. Data are shown as fold change relative to the TNF-α/IFN-γ-treated group and expressed as mean ± SEM from two independent experiments. ^##^ *p* < 0.01, ^###^ *p* < 0.01 vs. vehicle control; * *p* < 0.05, ** *p* < 0.01, *** *p* < 0.001 vs. TNF-α/IFN-γ-treated group.

At the mRNA level, TNF-α/IFN-γ stimulation resulted in a significant increase in *IL-1β* mRNA expression, with levels rising to 6.84 ± 0.64-fold relative to the untreated control group (*p* < 0.001) (Figure 4A). Harringtonine co-treatment significantly reduced this TNF-α/IFN-γ-induced increase in *IL-1β* mRNA expression, lowering it to 4.78 ± 0.16-fold at 3 μM (*p* < 0.05), 2.65 ± 0.28-fold at 10 μM (*p* < 0.01) and 2.21 ± 0.75-fold at 30 μM (*p* < 0.01).

Similarly, *IL-6* mRNA expression was markedly elevated following TNF-α/IFN-γ treatment, with expression levels reaching 7.54 ± 0.65-fold compared to the control group (*p* < 0.001) (Figure 4B). Harringtonine co-treatment significantly decreased *IL-6* mRNA expression to 6.18 ± 0.45-fold at 10 μM (*p* < 0.05) and 3.25 ± 0.15-fold at 30 μM (*p* < 0.01) compared to the TNF-α/IFN-γ-treated group.

*IL-8* mRNA expression also showed a significant increase in response to TNF-α/IFN-γ stimulation, reaching 8.65 ± 0.18-fold compared to the control group (*p* < 0.01) (Figure 4C). Co-treatment with harringtonine significantly suppressed this increase, reducing *IL-8* mRNA expression to 7.15 ± 0.55-fold at 3 μM (*p* < 0.05), 7.08 ± 0.91-fold at 10 μM (not significant) and 4.56 ± 0.48-fold at 30 μM (*p* < 0.01) compared to the TNF-α/IFN-γ-only group.

Treatment with TNF-α/IFN-γ (20 ng/mL) significantly increased IL-1β secretion in NHEKs compared to the untreated control group, with the secretion level reaching 35.97 ± 0.95 ng/mL (*p* < 0.001) (Figure 4D). However, co-treatment with harringtonine prior to TNF-α/IFN-γ stimulation significantly reduced IL-1β secretion, lowering it to 30.49 ± 2.49 ng/mL at 3 μM (not significant), 12.51 ± 3.47 ng/mL at 10 μM (*p* < 0.01) and 16.04 ± 4.80 ng/mL at 30 μM (*p* < 0.01) compared to the TNF-α/IFN-γ-only group.

Similarly, TNF-α/IFN-γ stimulation markedly elevated IL-6 secretion to 59.15 ± 2.59 ng/mL, showing a significant increase compared to the control group (*p* < 0.001) (Figure 4E). This increase in IL-6 secretion was significantly attenuated by harringtonine co-treatment, with levels reduced to 32.15 ± 1.84 ng/mL at 10 μM (*p* < 0.01) and 12.80 ± 4.51 ng/mL at 30 μM (*p* < 0.001) compared to the TNF-α/IFN-γ-treated group.

In the case of IL-8, its secretion level was also significantly increased after TNF-α/IFN-γ treatment, reaching 80.84 ± 3.08 ng/mL compared to the control (*p* < 0.001) (Figure 4F). Harringtonine co-treatment effectively reduced IL-8 secretion to 65.06 ± 6.14 ng/mL at 3 μM (*p* < 0.05), 32.64 ± 1.84 ng/mL at 10 μM (*p* < 0.01) and 32.10 ± 9.25 ng/mL at 30 μM (*p* < 0.01) compared to the TNF-α/IFN-γ-only group.

### 3.4. Effect of Harringtonine on the Secretion of PGE_2_, COX-2, and NO in TNF-α/IFN-γ-Stimulated NHEKs

The secretion levels of the inflammatory mediators PGE_2_, COX-2, and NO were quantitatively analyzed to evaluate the anti-inflammatory effects of harringtonine in TNF-α/IFN-γ-stimulated NHEKs.

**Figure 5:**
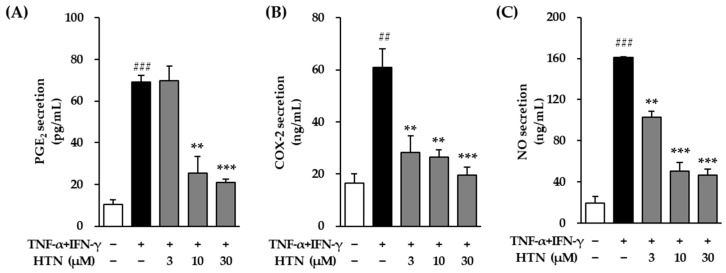
Effects of harringtonine (HTN) on protein secretion of PGE_2_, COX-2, and NO in normal human epidermal keratinocyte (NHEKs). NHEKs were seeded at 2 × 10^4^ cells/well in 48-well plates and incubated for 24 h. After serum starvation for 24 h, the cells were pretreated with harringotnine for 1 h, followed by stimulation with 20 ng/mL TNF-α/IFN-γ for 24 h. Secretion of PGE_2_ (**A**), COX-2 (**B**), and NO (**C**) was quantified using ELISA and the Griess assay. Data are shown as a fold change relative to the TNF-α/IFN-γ-treated group and expressed as mean ± SEM from two independent experiments. ^##^ *p* < 0.01, ^###^ *p* < 0.001 vs. vehicle control; ** *p* < 0.01, *** *p* < 0.001 vs. TNF-α/IFN-γ-treated group.

Treatment with TNF-α/IFN-γ (20 ng/mL) for 24 h significantly increased PGE_2_ secretion in NHEKs compared to the untreated control group, with the secretion level reaching 69.10 ± 3.07 ng/mL (*p* < 0.001) (Figure 5A). However, co-treatment with harringtonine prior to TNF-α/IFN-γ stimulation significantly reduced PGE_2_ secretion, lowering it to 25.45 ± 8.08 ng/mL at 10 μM (*p* < 0.01) and 21.09 ± 1.56 ng/mL at 30 μM (*p* < 0.001) compared to the TNF-α/IFN-γ-only group (*p* < 0.01).

Similarly, TNF-α/IFN-γ stimulation markedly elevated COX-2 secretion to 61.01 ± 7.15 ng/mL, showing a significant increase compared to the control group (*p* < 0.01) (Figure 5B). This increase in COX-2 secretion was significantly attenuated by harringtonine co-treatment, with levels reduced to 28.21 ± 6.30 ng/mL at 3 μM (*p* < 0.01), 26.57 ± 2.58 ng/mL at 10 μM (*p* < 0.01) and 19.50 ± 3.14 ng/mL at 30 μM (*p* < 0.001) relative to the TNF-α/IFN-γ-treated group.

In the case of NO, its secretion level was also significantly increased after TNF-α/IFN-γ treatment, reaching 160.69 ± 1.05 ng/mL compared to the control (*p* < 0.001) (Figure 5C). Harringtonine co-treatment effectively reduced NO secretion to 102.62 ± 6.14 ng/mL at 3 μM (*p* < 0.01), 50.07 ± 8.84 ng/mL at 10 μM (*p* < 0.001) and 46.31 ± 6.03 ng/mL at 30 μM (*p* < 0.001) relative to the TNF-α/IFN-γ-treated group.

## 4. Discussion

In this study, we comprehensively investigated the protective effects of harringtonine, an alkaloid isolated from *C. harringtonia*, against skin aging and inflammation in normal human epidermal keratinocytes (NHEKs) exposed to combined stimulation with TNF-α and IFN-γ, two pivotal cytokines involved in skin inflammatory responses. Our findings demonstrate that harringtonine exerts significant multi-targeted actions, influencing extracellular matrix (ECM) homeostasis, skin barrier function, and inflammatory cytokine production, thereby providing compelling evidence of its potential as a therapeutic candidate for mitigating skin damage induced by pro-inflammatory stress.

First, harringtonine markedly suppressed the expression of matrix metalloproteinases (MMP-1, MMP-2, and MMP-9), which are critical mediators of ECM degradation and play central roles in the pathogenesis of skin aging and photoaging [14]. By attenuating TNF-α/IFN-γ-induced upregulation of these enzymes, harringtonine may effectively prevent collagen breakdown and dermal matrix disorganization, thereby preserving skin structural integrity. Furthermore, harringtonine partially restored the expression of collagen synthesis-related genes, including *COL1A1*, *COL1A2*, and *COL4A1*, which were significantly downregulated under inflammatory conditions. The restoration of these genes suggests that harringtonine not only inhibits collagen degradation but also promotes collagen biosynthesis, potentially contributing to ECM regeneration and skin repair processes [15].

In addition to its effects on ECM remodeling, harringtonine exhibited a pronounced ability to ameliorate the downregulation of skin barrier-associated genes such as *SPINK5*, *LOR*, *AQP3*, *FLG*, and *KRT1*. These genes are essential for maintaining epidermal barrier integrity, hydration, and protection against external factors [16,17]. The restoration of these genes by harringtonine implies that it may help reinforce the skin barrier, thereby enhancing the skin’s resilience to environmental stressors and inflammation. Interestingly, while harringtonine effectively restored several skin barrier-related genes, it did not significantly affect *IVL* expression, suggesting that its regulatory effects may vary depending on the specific gene or signaling context of the study. This selective modulation warrants further investigation to elucidate its underlying mechanisms.

Moreover, harringtonine exhibited strong anti-inflammatory activity by significantly reducing the mRNA expression and protein secretion of key pro-inflammatory cytokines, including IL-1β, IL-6, and IL-8, which are known to exacerbate skin inflammation and aging. These cytokines not only promote local inflammation but also contribute to ECM degradation and skin barrier impairment [18,19,20]. Notably, the inhibitory effects of harringtonine were dose-dependent, with higher concentrations exhibiting stronger suppression of cytokine expression and secretion than lower concentrations.

Beyond cytokine modulation, harringtonine also attenuates the production of inflammatory mediators, such as prostaglandin E_2_ (PGE_2_), cyclooxygenase-2 (COX-2), and nitric oxide (NO), all of which are implicated in chronic inflammation and oxidative stress within the skin microenvironment. The suppression of these mediators by harringtonine suggests that it may exert broader anti-inflammatory and antioxidative effects, thereby interrupting the multiple pathological pathways involved in skin aging.

Harringtonine has been previously investigated for its wide range of pharmacological activities, including anti-cancer, anti-inflammatory, and antileukemic activities [12,21,22]. It has been shown to induce apoptosis and inhibit proliferation in various cancer cell types by targeting key signaling pathways, such as NF-κB, STAT3, and MAPKs [12]. These established bioactivities support its therapeutic potential beyond oncology, suggesting that its capacity to modulate inflammation, ECM remodeling, and fibrosis-related pathways may be broadly applicable to chronic inflammatory skin conditions and age-related skin deterioration.

Collectively, these results highlight the potential of harringtonine as a multifunctional agent capable of simultaneously targeting ECM degradation, skin barrier dysfunction, and inflammation. Its ability to modulate diverse molecular pathways suggests that it can be developed as a comprehensive therapeutic option for preventing or ameliorating skin aging and inflammatory skin diseases. Furthermore, considering the complexity of skin aging, which involves a dynamic interplay between oxidative stress, chronic inflammation, and structural damage, agents such as harringtonine that exhibit multi-target effects are particularly valuable.

However, several questions remain to be addressed. While the current study provides robust in vitro evidence using keratinocytes, it is essential to validate the protective effects of harringtonine in in vivo models of skin aging or inflammation to fully assess its therapeutic potential and safety profile. Future research should explore the precise molecular mechanisms by which harringtonine modulates these diverse pathways, including its potential interactions with key signaling cascades, such as NF-κB, MAPKs, and STATs.

These findings collectively highlight harringtonine’s potential as a multifunctional therapeutic candidate targeting key pathways involved in skin inflammation and aging. The observed protective effects against TNF-α/IFN-γ-induced extracellular matrix degradation, skin barrier disruption, and cytokine overproduction suggest its suitability for topical application in cosmetic or therapeutic settings. In particular, its broad mechanism of action supports further investigation into harringtonine as a lead compound for the development of anti-aging or anti-inflammatory dermatological formulations.

However, this study was conducted exclusively in an in vitro keratinocyte model, which, while valuable for elucidating cellular mechanisms, does not fully recapitulate the complexity of skin tissue in vivo. Therefore, further validation using three-dimensional skin equivalents and in vivo models is essential to evaluate the pharmacodynamic properties, safety, and skin penetration capacity of harringtonine. These future studies will help bridge the gap between in vitro findings and clinical translation, ultimately determining the practical utility of harringtonine in real-world dermatological applications.

In conclusion, our study provides compelling evidence that harringtonine alleviates TNF-α/IFN-γ-induced skin damage by targeting multiple aspects of skin aging and inflammation, including ECM remodeling, skin barrier function, and inflammatory cytokine production. These findings suggest that harringtonine is a promising natural bioactive compound for use in anti-aging skincare formulations or therapeutic interventions for inflammatory skin conditions. Further mechanistic and translational studies are required to validate its clinical applicability.

## 5. Conclusions

In this study, we systematically investigated the protective effects of harringtonine on TNF-α/IFN-γ-induced skin inflammation and aging in NHEKs. Our findings demonstrate that harringtonine acts as a multifunctional agent by modulating key molecular mechanisms involved in skin aging. Harringtonine effectively suppressed MMPs (*MMP-1*, *MMP-2*, and *MMP-9*), preserving ECM integrity by preventing collagen degradation and enhancing the expression of collagen synthesis-related genes (*COL1A1*, *COL1A2*, and *COL4A1*). It also improved the expression of skin barrier-related genes (*SPINK5*, *LOR*, *AQP3*, *FLG*, and *KRT1*), although *IVL* expression was not significantly affected by the treatment. Additionally, harringtonine exhibited potent anti-inflammatory effects by reducing both the mRNA expression and secretion of pro-inflammatory cytokines (IL-1β, IL-6, and IL-8) and inflammatory mediators, such as PGE_2_, COX-2, and NO. Collectively, these results highlight the comprehensive protective effects of harringtonine against skin aging and inflammation through the regulation of ECM remodeling, skin barrier function, and inflammatory responses. Our findings suggest the potential of this compound as an active ingredient in anti-aging skincare formulations or therapeutic interventions for inflammatory skin conditions. Further in vivo studies and mechanistic investigations are warranted to confirm the clinical applicability and safety of this approach.

## Figures and Tables

**Table 1:** Primer sequences.

Gene	Sense Primer Sequence (5′-3′)	Antisense Primer Sequence (5′-3′)
*MMP-1*	ATTCTACTGATATCGGGGCTTT	ATGTCCTTGGGGTATCCGTGTA
*MMP-2*	CAGGGAATGAGTACTGGGTCTATT	ACTCCAGTTAAAGGCAGCATCTAC
*MMP-9*	CACTGTCCACCCCTCAGAGC	CACTTGTCGGCGATAAGG
*COL1A1*	CTCGAGGTGGACACCACCCT	CAGCTGGATGGCCACATCGG
*COL1A2*	AGAAACACGTCTGGCTAGGAG	GCATGAAGGCAAGTTGGGTAG
*COL3A1*	GTTTTGCCCCGTATTATGGA	GGAAGTTCAGGATTGCCGTA
*COL4A1*	ACTCTTTTGTGATGCACACCA	AAGCTGTAAGCGTTTGCGTA
*HAS-1*	CCACCCAGTACAGCGTCAAC	CATGGTGCTTCTGTCGCTC
*HAS-2*	TTTGTTCAAGTCCCAGCAGC	ATCCTCCTGGGTGGTGTGAT
*HAS-3*	CCCAGCCAGATTTGTTGATG	AGTGGTCACGGGTTTCTTCC
*SPINK5*	CTGCTCCATGTGTGAGGTCTTC	CGTCCGTTTTTCCTGGATTTGCG
*LOR*	GTGGGAGCGTCAAGTACTCC	AGAGTAGCCGCAGACAGAGC
*IVL*	CAACTGGAGCTCCCAGAGCAGC	AACACAGGCTGCTCCAGCTGC
*AQP3*	TCTTTGACCAGTTCATAGGCAC	GGCAGGGTTGACGGCATAG
*FLG*	GCTGAAGGAACTTCTGGAAAAGG	GTTGTGGTCTATATCCAAGTGATC
*KRT1*	CTGGCAGACATGGGGATAGTGTG	CTGATGGTGGTGTGGCTTGTGCT
*IL-1β*	AGCTACGAATCTCCGACCAC	CGTTATCCCATGTGTCGAAGAA
*IL-6*	ACTCACCTCTTCAGAACGAATTG	CCATCTTTGGAAGGTTCAGGTTG
*IL-8*	GAGAGTGATTGAGAGTGGACCAC	CACAACCCTCTGCACCCAGTTT
*β-actin*	AGAGATGGCCACGGCTGCTT	ATTTGCGGTGGACGATGGAG

## Data Availability

This published article contains all the data created or analyzed during this study. Any additional data or information can be made available to the corresponding author upon request.

## Supplementary Materials

The following supporting information can be downloaded at: https://www.mdpi.com/article/10.3390/cimb47080642/s1.

## Abbreviations

The following abbreviations are used in this manuscript:

AQP3	Aquaporin 3
COL1A1	Collagen Type I Alpha 1 Chain
COL1A2	Collagen Type I Alpha 2 Chain
COL3A1	Collagen Type III Alpha 1 Chain
COL4A1	Collagen Type IV Alpha 1 Chain
COX-2	Cyclooxygenase-2
ECM	Extracellular Matrix
FLG	Filaggrin
HAS-1	Hyaluronic Acid Synthase 1
HAS-2	Hyaluronic Acid Synthase 2
HAS-3	Hyaluronic Acid Synthase 3
HTN	Harringtonine
IFN-γ	Interferon-gamma
IL-1β	Interleukin-1 beta
IL-6	Interleukin-6
IL-8	Interleukin-8
IVL	Involucrin
JNK	c-Jun N-terminal Kinase
KRT1	Keratin 1
LOR	Loricrin
MAPKs	Mitogen-Activated Protein Kinases
MMP-1	Matrix Metalloproteinase-1
MMP-2	Matrix Metalloproteinase-2
MMP-9	Matrix Metalloproteinase-9
NHEKs	Normal Human Epidermal Keratinocytes
NO	Nitric Oxide
PGE_2_	Prostaglandin E_2_
ROS	Reactive Oxygen Species
SPINK5	Serine Peptidase Inhibitor Kazal Type 5
TNF-α	Tumor Necrosis Factor-alpha
UV	Ultraviolet
