# Supplementary material for: Harringtonine Attenuates Extracellular Matrix Degradation, Skin Barrier Dysfunction, and Inflammation in an In Vitro Skin Aging Model
Source: Curr Issues Mol Biol. 2025 Aug 10;47(8):642. doi: 10.3390/cimb47080642 (PMC12385131; doi:10.3390/cimb47080642)
Supplement: Supplementary file 1 [file cimb-47-00642-s001.zip › cimb-3783627-supplementary.pdf]

Single Injection Report

Line#	Inj#	Location	Sample Name	Sample Amt	Multiplier * Dilution	Datafile	Cal Lvl
6	1	P1-B2	805 1000		1.0000	805 1000.dx	6

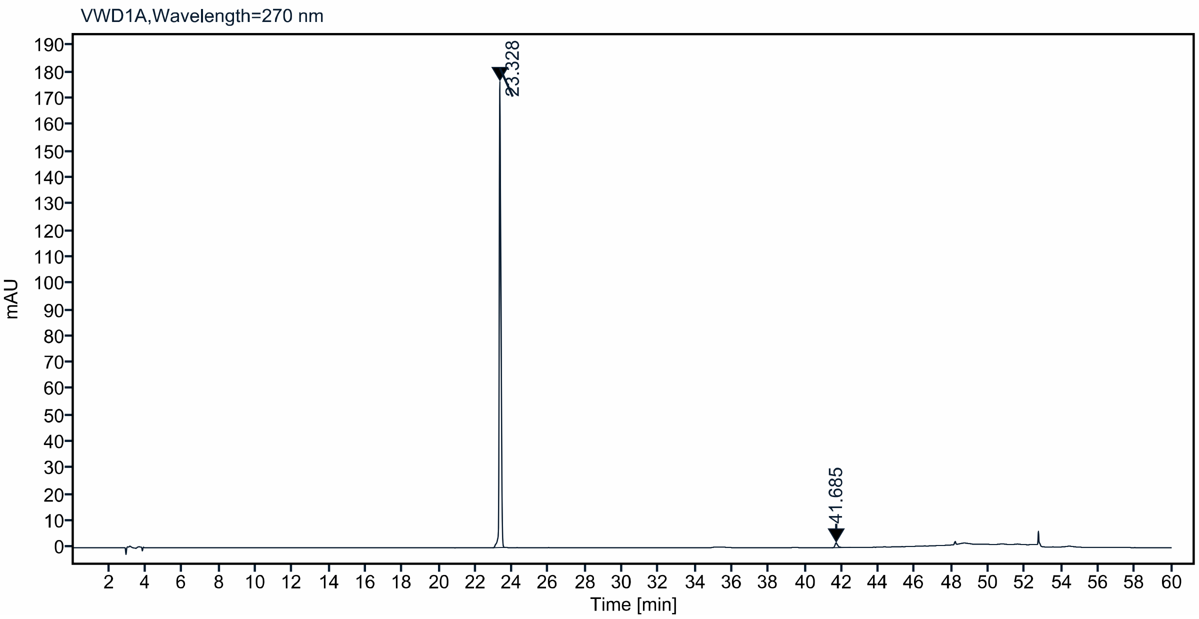

Signal: VWD1A,Wavelength=270 nm

RT [min]	Type	Width [min]	Area	Height	Area%	Name
23.328	BB	0.7050	1086.3883	176.5486	98.2855	
41.685	BB	1.0183	18.9505	1.8071	1.7145	
		**Sum**	**1105.3388**			
